# Challenges to and Countermeasures for the Value Realization of Healthcare Data Elements in China

**DOI:** 10.1002/hcs2.70015

**Published:** 2025-05-15

**Authors:** Tianan Yang, Wenhao Deng, Ran Liu, Tianyu Wang, Yuanyuan Dai, Jianwei Deng

**Affiliations:** ^1^ School of Management Beijing Institute of Technology Beijing China; ^2^ Sustainable Development Research, Institute for Economy and Society of Beijing Beijing Institute of Technology Beijing China; ^3^ Yangtze River Delta Research Institute Beijing Institute of Technology Jiaxing Zhejiang China; ^4^ School of Medical Humanities and Management Wenzhou Medical University Wenzhou Zhejiang China; ^5^ Key Research Center of Philosophy and Social Sciences of Zhejiang Province (Institute of Medical Humanities) Wenzhou Medical University Wenzhou Zhejiang China; ^6^ Henley Business School University of Reading Reading UK

**Keywords:** China, healthcare data elements, healthcare data management, value realization

## Abstract

The market for healthcare data elements in China has shown strong growth, but still fails to effectively realize the value of circulation and transactions. By identifying the five major challenges, this article proposes countermeasures to accelerate the release of multidimensional value and integrative value creation of healthcare data elements.

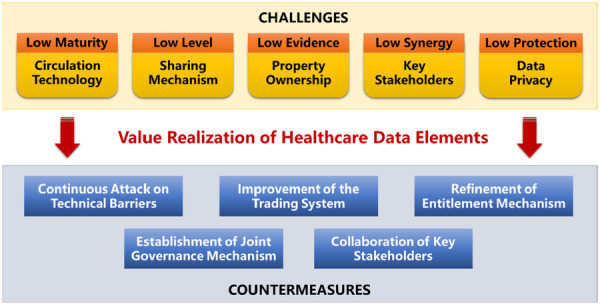

## 1 Introduction

**Figure 1:**
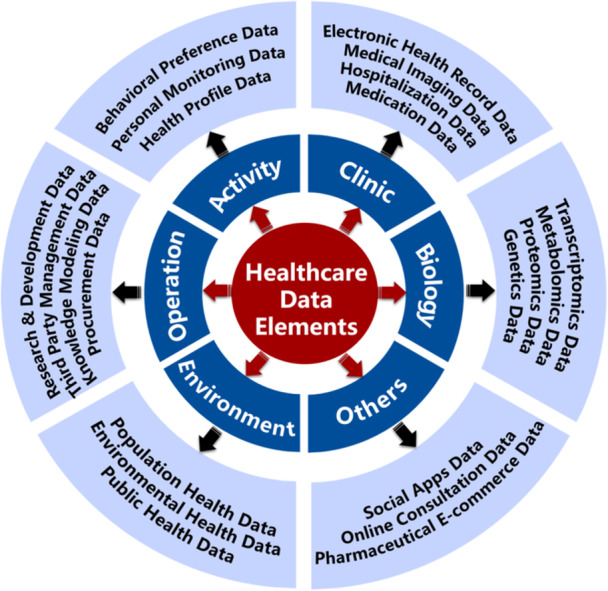
Essential components of healthcare data elements.

As a new type of production factor in healthcare, healthcare data elements have been rapidly integrated into various health production processes, such as clinical assistance, health management, biological testing, and operation and supervision [1, 2]. Healthcare data elements include biological and clinical data that are related to disease, environmental health data that are associated with life, and operational and healthcare management data that are related to healthcare activities (Figure 1). Activities such as the construction of a data value assessment system, the development of a data circulation and sharing platform, and the authorization of data compliance and operation products support the strong growth momentum of the market for healthcare data elements in China [3].

**Figure 2:**
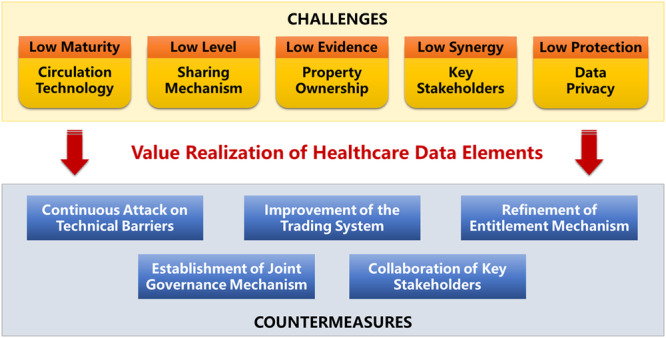
Challenges to and countermeasures for realizing value from healthcare data elements.

Although healthcare data in China are not lacking, effectively realizing the circulation and transaction value of a substantial number of healthcare data elements remains difficult given the limited investment in and attention to data technology development, trusted circulation, and security regulation. To support achieving the *Global Strategy on Digital Health 2020–2025* [4], this article summarizes the challenges to further driving the value realization of healthcare data elements in China and proposes countermeasures (Figure 2).

## 2 Challenges in Realizing Value of Healthcare Data Elements

### 2.1 Low Maturity of Circulation Technology

Healthcare data elements are associated with multidimensional issues related to sensitivity and complexity, requirity mature technologies to support the safe flow of healthcare data among multiple subjects [5]. In 2023, the healthcare industry in China leaked more than 900 million pieces (or roughly 344.7 GBs) of data. Given the differences in the format, structure, and coding of healthcare data, the difficulty in creating data interfaces, and the lack of unified technical specifications for healthcare information systems, data security issues caused by immature technology have become the primary challenge to the circulation of healthcare data elements in China.

### 2.2 Limited Sharing Mechanisms

The value of healthcare data elements lies in their application, and the essence of application lies in compatibility and sharing [6]. For example, Siemens Healthcare in Germany has cooperated with hospitals in China to build the “Meta‐Universe Application Scenario of Medical Digital Human” project, which accelerates the clinical application of technologies such as digital twins, the Internet of Things, and brain‐computer interfaces and shares and applies healthcare data elements in various scenarios. However, the lack of quality control management and standardized tools for healthcare data elements has led to inconsistencies in the development and implementation of data sharing and open standards, making it difficult to transform substantial amounts of data into the high‐value knowledge required for diverse scenarios and knowledge‐supported decision‐making.

### 2.3 Limited Evidence of Property Ownership

Although the healthcare data element system in China has actively explored the definitions of ownership, classification and grading, and revenue distribution, effective incentives, supervision, and rights protection mechanisms remain lacking. The *Health Insurance Portability and Accountability Act* [7] proposed by the United States has become an important international guideline for protecting health privacy and security. For example, in Aliyun's (Alibaba Cloud Computing Co Ltd, Hangzhou, China) cooperation with Japanese enterprises on artificial intelligence‐based diagnostic technology services, cloud service providers' responsibilities in health data protection, compliance, and risk assessment were clarified in accordance with this act. In contrast, most of the stakeholders of healthcare data in China pay less attention to the utilization and development of healthcare data elements and have yet to establish specific rights management methods or assessment and audit standards.

### 2.4 Low Synergy of Key Stakeholders

Realizing the value of human‐centered healthcare data elements requires the engagement of potential stakeholders—including patients, families, communities, service providers, policymakers, and manufacturers—along the value chain. For example, scholars in the United States have taken a broad stakeholder‐engaged approach to clinical data collection and have successfully constructed viable, reliable, and valid eClinical quality metrics as a way of avoiding major and potentially unprofitable investments in resources [8]. More than 10 colleges and universities in China have established disciplines related to healthcare data to accelerate addressing the shortage of human resources in healthcare. Considering the relatively strict data policy control in China, most healthcare data sharing and circulation is directly decided by the government administration and the decision‐making level of core hospitals. Therefore, the interests of data processors and users (e.g., patients, medical staff, and third‐party organizations) are difficult to satisfy, limiting stakeholders' interest in linking efforts and their willingness to implement policies.

### 2.5 Low Protection of Data Privacy

The challenge of healthcare data privacy exists in each of the above‐mentioned aspects of technological upgrading, data sharing, and collaboration. However, ensuring data privacy is a fundamental guarantee for realizing the value of healthcare data elements. The European Commission has taken the lead in funding the “HealthyCloud” initiative, which mandates the construction of a robust and consistent healthcare data governance approach to address the ethical and legal issues associated with the collection and use of healthcare data elements [9]. The acquisition, use, or control of healthcare data elements by malicious competitors could lead to the loss of advantages in medical technology, drug development, disease prevention, and data control or to the threat of attacks such as bioterrorism. Additionally, the improper use of healthcare data elements may cause an increase in healthcare fraud and medical disputes, thus damaging patient trust and public safety. Therefore, the difficulty of balancing the need for privacy protection and the promotion of healthcare innovation is a significant barrier to ensuring data security and compliance.

## 3 Countermeasures for Realizing the Value of Healthcare Data Elements

### 3.1 Continuous Attack on Technical Barriers

Breaking through the limitations of traditional production approaches in the healthcare industry requires balancing the privacy and security protection of healthcare data elements. The “14th 5‐Year Plan” for national health informatization requires accelerating research on data integration and insight technology to extract useful information from a substantial amount of healthcare data to provide a more accurate basis and guidance for decision‐making. According to this, the scale of technology application, including supporting healthcare institutions, enterprise organisations, and other subjects must be strengthened to apply healthcare data circulation technology. Wuzhen in China launched an open platform for healthcare data applications by leveraging the advantages of arithmetic power and artificial intelligence technology to generate a “healthy digital person” application for each platform user. The application includes information on physical signs and symptoms, medical care, health insurance, and medicine. In addition, the technical compliance regulatory system must be improved to continuously strengthen the regulatory governance of unfair competition behaviors such as monopoly, falsification, leakage, and abuse of healthcare data.

### 3.2 Improvement of the Trading System

Combining healthcare data elements with emerging production application scenarios is an essential path for achieving data sharing and collaborative governance across business lines. First, a management system for healthcare data trading venues must be developed and the healthcare data trading processes, such as trade application, negotiation, and implementation must be standardized. As part of this process, a transaction data protection system that strictly follows the principles of lawful compliance, legitimacy, and necessity must be established to avoid unethical use or misuse. Second, conducting specialized research on standardization in the field of healthcare data elements and promoting pilot projects must be accelerated. Third, fostering third‐party professional organizations for healthcare data integration, compliance certification, security auditing, data notarization, data insurance, and risk assessment can improve the ability to provide full‐process services for the circulation and trading of healthcare data elements. In 2023, Hangzhou authorized AliHealth Technology Co. (Alibaba Health Technology Co Ltd, Beijing, China) to conduct a 2‐year public data authorization operation to open up multiple health service application scenarios.

### 3.3 Refinement of Entitlement Mechanism

Clarifying the ownership of property rights must incentivize and safeguard a consensus on the rights of stakeholders in data development and utilization. First, exploring a mechanism for the distribution of proceeds from multiple types of healthcare data requires the establishment of a public healthcare data revenue feedback mechanism. Second, it is important to support the exploration of diversified pricing models and price formation mechanisms by playing a guiding or autonomous decision‐making role in authorizing the use of different types of data by the government and the market. The first real healthcare data pricing model in China has relied on a healthcare data circulation and privacy computing solution provider to build a new ecology of real healthcare collaborators [10]. Third, clarification of the rules for healthcare data collection, processing, use, and operation by scenario is required. In conjunction with several departments, the National Health Commission has committed to issuing the *Guidelines on Data Classification and Grading for the Health Industry* [11], which will establish a catalog system for health and medical data resources to itemize data content, scale, and application scenarios. Off‐site trading should be supported for healthcare data trading activities for which clear responsibilities, rights, and simple circulation methods are outlined and which do not involve personal privacy or state secrets.

### 3.4 Establishment of Joint Governance Mechanism

Establishing a coordinated mechanism to advance the healthcare data elements market requires collaborating between relevant departments at all levels and assuming overall responsibility for nurturing the healthcare data elements market is necessary. The National Data Bureau and other departments in China jointly issued a 3‐year action plan regarding data elements that help public medical institutions share data with financial, pension, and other business entities and strengthen the integration of healthcare data with innovative new forms of joint governance. Optimizing the governance responsibilities for healthcare data elements and clarifying the division of functions requires the timely establishment of a data supervision committee and the exploration of a collaborative regulatory mechanism. However, relying on the support of an efficient, coordinated, interconnected, centralized, and integrated data infrastructure platform requires the gradual clarification of the roles of investors, authorities, and enablers in the governance of healthcare data elements.

### 3.5 Collaboration of Key Stakeholders

Given the multiple interests and the complex division of responsibilities, the rights and interests of all stakeholders must be protected to motivate these stakeholders to actively cooperate. First, platforms for mutual trust and cooperation with clear rules must be established. For example, Wenzhou has been at the forefront of promoting the utilization of public data resources in healthcare by building a data “safe harbor” consisting of legal protection, talent, facilities, and funds. Second, the accurate collection, integration, and collaboration of healthcare data elements must be ensured by providing cross‐disciplinary training of human resources in healthcare**—**a core resource for ensuring the high‐quality development of healthcare data elements in this technology‐intensive system [12]. Finally, public opinion must be widely solicited and mobilized while respecting citizens' healthcare rights (e.g., privacy and autonomy). Given the disadvantages in access, ability, and willingness among underserved groups [13], more convenient channels of participation and feedback should be opened, and patient trust in healthcare data elements should be built from the grassroots level to bridge any healthcare data divide that may exist.

## 4 Conclusion

Overall, tens of billions of healthcare data elements in China have become fundamental strategic resources for the development of new‐quality productive forces. By identifying the five major challenges and dilemmas in realizing the value of healthcare data elements, this article emphasizes that government departments and all sectors in society must continuously improve policies to develop healthcare data elements, strive to promote the marketization and industrialization of healthcare data elements and accelerate the realization of multidimensional value and the dynamic and integrated value creation from healthcare data elements.

## Author Contributions

**Tianan Yang:** funding acquisition (equal); writing – original draft (lead); writing – review and editing (equal). **Wenhao Deng:** conceptualization (lead); writing – original draft (equal); writing – review and editing (lead). **Ran Liu:** investigation (lead); writing – original draft (supporting). **Tianyu Wang:** visualization (lead); writing – review and editing (supporting). **Yuanyuan Dai:** investigation (supporting); writing – review and editing (supporting). **Jianwei Deng:** funding acquisition (equal); supervision (lead).

## Ethics Statement

The authors have nothing to report.

## Consent

The authors have nothing to report.

## Conflicts of Interest

The authors declare no conflicts of interest.

## Data Availability

The authors have nothing to report.
